# Auricular perichondritis by piercing complicated with pseudomonas infection

**DOI:** 10.1016/S1808-8694(15)31034-X

**Published:** 2015-10-19

**Authors:** Felipe Montes Pena, Daniela Mendonça Sueth, Maria Irene Rocha Bastos Tinoco, Janine Franklin Machado, Luiz Eduardo O. Tinoco

**Affiliations:** a Student.; b Medical Student.; c Dermatologist.; d Medical Student.; e MD. Otorhinolaryngologist. Hospital São José do Avaí.

**Keywords:** perichondritis, piercing, pseudomonas infection

## INTRODUCTION

Perichondritis is a slow evolving infection, located on the ear cartilage. They result from lacerations, blunt trauma, surgeries and other affections. Ear piercing induced pinna perichondritis is a frequent affection among the young population. The most commonly found etiologic agent is Pseudomonas aeruginosa and its inoculation caused by pinna cartilage and pericondrium exposure during or after the piercing procedure. Etiologic investigation is mandatory by means of secretion culture and antibiogram. Treatment is based on surgical drainage, systemic antibiotics and anti-inflammatory agents.^1^

## CASE REPORT

**Figure 1:**
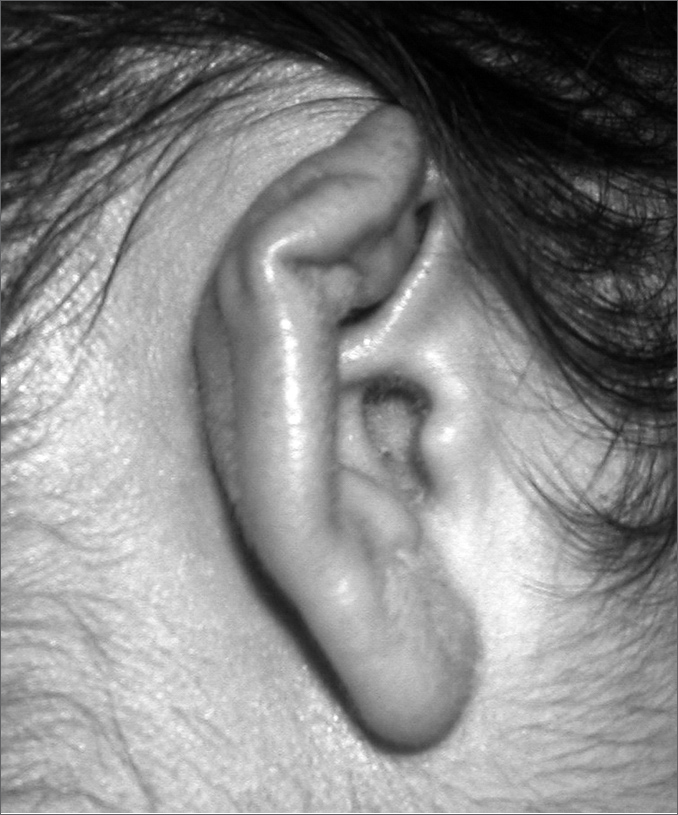
Post-treatment aspect with a view of the pinna.

A.F.C., 26 years of age, White, married, Born in Itaperuna-RJ, reports having pierced her ear cartilage and after eight days started having pain, local edema and hyperemia. She used potassium diclofenac, without improvement. Fourteen days later she went to see an otorhinolaryngologist, who diagnosed an abscess and decided to drain it and start her on antibiotics with cephalexin and prednisone. The wound did not respond well, and the patient needed urgent care with intravenous analgesic agents and, after one week, a new drainage procedure was carried out. At this visit, material for culture was harvested, which was positive for Pseudomonas aeruginosa, with immediate start of ciprofloxacin and new drainage, with improvement. She then evolved with scar deformity and has been submitted to other treatments in order to enhance scar appearance (Figure 1).

## DISCUSSION

Body piercing is known as a tribal culture in some countries of Africa and Arabia, and commonly worn in the nose, lips and tongue.^2^

The description of a first Pseudomonas aeruginosa causing perichondritis after piercing is somewhat recent. It usually happens in warm months, when body sweating is excessive, impairing healing and predisposing the site to infections. The risk of developing an infection is higher in the ear cartilage than it is in the ear lobe^3^.

Symptoms start in a few hours, and three days after the piercing takes place, characterized by site hardening, redness, pain during palpation and blood or pus oozing for fourteen months or more.^3^ The most commonly found etiologic agent is Pseudomonas aeruginosa, and this affection is less commonly caused by Staphylococcus aureus.

Diagnosis is essentially clinical, when secretion culture and antibiogram must be performed. A broad surgical incision and necrotic tissue removal is Paramount for the healing process to take place, aiming at precluding deformities. Clinical treatment is based on the use antimicrobial agents such as cephalosporin and quinolone, in order to eradicate Pseudomonas aeruginosa.1 Major complications are contact dermatitis and hypersensitivity reaction towards nickel.4 The most frequent sequela is pinna deformity that assumes a cauliflower aspect.^1^

Some studies show that pinna piercing is a risk factor for Hepatitis B transmission.^5^ Non-infectious problems, hypersensitivity to metals, may be even more common than a serious infection.^6^ Piercing-caused perichondritis presents large morbidity, and it evolved towards complications in up to 35% of the patients.^4^

We then conclude that body piercing brings about adverse reactions such as secondary infections and the risk of acquiring B hepatitis and AIDS. The pinna cartilage piercing has a greater risk of infection, and in pinna chondritis Pseudomonas aeruginosa must be considered as the probable cause of the disease. Although post-treatment results are good, cosmetic deformities may happen, and are more evident the later proper treatment is installed.
